# Ranking genomic features using an information-theoretic measure of epigenetic discordance

**DOI:** 10.1186/s12859-019-2777-6

**Published:** 2019-04-08

**Authors:** Garrett Jenkinson, Jordi Abante, Michael A. Koldobskiy, Andrew P. Feinberg, John Goutsias

**Affiliations:** 1 Whitaker Biomedical Engineering Institute, Johns Hopkins University, Baltimore, MD USA; 2 Center for Epigenetics, Johns Hopkins School of Medicine, Baltimore, MD USA; 3 Currently with Department of Health Sciences Research, Mayo Clinic, Rochester, MN USA; 4 Pediatric Oncology, Sidney Kimmel Comprehensive Cancer Center, Johns Hopkins University School of Medicine, Baltimore, MD USA; 5 Department of Biomedical Engineering, Johns Hopkins University, Baltimore, MD USA; 6 Department of Medicine, Johns Hopkins School of Medicine, Baltimore, MD USA

**Keywords:** DNA methylation, Genomic feature analysis, Information theory, Mutual Information, Gene ranking, Methylation analysis, WGBS data analysis

## Abstract

**Background:**

Establishment and maintenance of DNA methylation throughout the genome is an important epigenetic mechanism that regulates gene expression whose disruption has been implicated in human diseases like cancer. It is therefore crucial to know which genes, or other genomic features of interest, exhibit significant discordance in DNA methylation between two phenotypes. We have previously proposed an approach for ranking genes based on methylation discordance within their promoter regions, determined by centering a window of fixed size at their transcription start sites. However, we cannot use this method to identify statistically significant genomic features and handle features of variable length and with missing data.

**Results:**

We present a new approach for computing the statistical significance of methylation discordance within genomic features of interest in single and multiple test/reference studies. We base the proposed method on a well-articulated hypothesis testing problem that produces *p*- and *q*-values for each genomic feature, which we then use to identify and rank features based on the statistical significance of their epigenetic dysregulation. We employ the information-theoretic concept of mutual information to derive a novel test statistic, which we can evaluate by computing Jensen-Shannon distances between the probability distributions of methylation in a test and a reference sample. We design the proposed methodology to simultaneously handle biological, statistical, and technical variability in the data, as well as variable feature lengths and missing data, thus enabling its wide-spread use on any list of genomic features. This is accomplished by estimating, from reference data, the null distribution of the test statistic as a function of feature length using generalized additive regression models. Differential assessment, using normal/cancer data from healthy fetal tissue and pediatric high-grade glioma patients, illustrates the potential of our approach to greatly facilitate the exploratory phases of clinically and biologically relevant methylation studies.

**Conclusions:**

The proposed approach provides the first computational tool for statistically testing and ranking genomic features of interest based on observed DNA methylation discordance in comparative studies that accounts, in a rigorous manner, for biological, statistical, and technical variability in methylation data, as well as for variability in feature length and for missing data.

**Electronic supplementary material:**

The online version of this article (10.1186/s12859-019-2777-6) contains supplementary material, which is available to authorized users.

## Background

Epigenetics encompasses all cellular processes that lead to heritable changes in gene expression without modifying the DNA sequence. Uncovering the role of epigenetics in regulating a cell’s phenotype is a key problem in modern molecular biology and medicine with important implications for understanding and treating human diseases, such as cancer.

The most studied epigenetic mechanism in humans is DNA methylation. This process chemically marks the DNA by adding a methyl group (CH_3_) to individual cytosines located immediately adjacent to guanines (CpG sites). In humans, a targeted enzymatic machinery allows for the heritable transmission of these epigenetic marks from parent to progeny cells during cell division. It turns out that this dynamic mechanism is partially responsible for the developmental establishment and ongoing maintenance of cellular identity, as well as for many deleterious biological processes, such as aging and carcinogenesis [1–4].

Whole genome bisulfite sequencing (WGBS) provides a genome-wide assessment of DNA methylation patterns along the genome at a single-base resolution. For this reason, many computational methods have been proposed in the literature for the extraction and analysis of methylation information from this type of data [5, 6]. One such technique, known as informME (information-theoretic analysis of methylation), provides the most advanced capabilities available to-date for the modeling and analysis of DNA methylation from WGBS data [7, 8]. The method employs principles from statistical physics and information theory to build statistical models for WGBS data that provide accurate and insightful assessment of methylation information well beyond the one performed by other approaches. This is accomplished through genome-wide methylation analysis by modeling the DNA methylation state within regions of the genome using joint probability distributions computed directly from WGBS data, by quantifying stochasticity using the information-theoretic notion of normalized methylation entropy (NME), and by detecting methylation discordances in test/reference comparisons using the Jensen-Shannon distance (JSD) between joint methylation probabilities in test and reference samples. These methylation metrics go beyond mean-based analysis and have shown great promise when studying development, aging, and cancer [7].

A fundamental problem when analyzing WGBS data is linking the genome-wide results back to specific genomic features of interest (e.g., genes), and ranking these features based on their significance. This problem is commonly addressed by a procedure that first labels CpG sites or regions of the genome as being differentially methylated between a reference and a test sample and then quantifies their degree of overlap with features of interest [9, 10]. Unfortunately, this method is confounded by the variable lengths of most genomic features of interest. For example, by recognizing the importance of intragenic DNA in gene expression [11, 12], we may consider gene bodies as the genomic features of interest, whose length varies appreciably throughout the genome. In this case, any differentially methylated region (DMR) that is randomly placed on the DNA will more likely overlap with long gene bodies than short ones, and this will seriously skew the analysis. In addition to the previous issue, we have argued in [8] that there is a fundamental loss of power when performing genome-wide statistical analysis for DMR detection followed by scoring features of interest using DMR overlaps, or other DMR metrics, as compared to a targeted approach that scores features by focusing the statistical analysis on the features themselves. For this reason, we developed in [8] an approach for ranking genes based on observed methylation discordances within their promoter regions, determined by a fixed window centered at their transcription start sites (TSSs), and showed that it outperforms DMR overlap-based analysis. However, this ranking method has two critical weaknesses. First, it cannot appropriately handle genomic features with variable lengths, since a genomic region is scored by a *p*-value whose calculation depends on the region’s length. Although this is not a problem for promoter regions, which are often taken to be of fixed length, it excludes other genomic regions of interest, such as gene bodies, exons, introns, bivalent domains, and enhancers. In addition, our method calculates a *p*-value, which is used to score a genomic region, by computing multiple *p*-values within the region, which are then combined using Fisher’s method [13]. This approach however generates a combined *p*-value or score that cannot be trusted when evaluating statistical significance, since the individual *p*-values are not necessarily statistically independent, as required by Fisher’s method.

In this paper, we introduce a new statistical approach for ranking genomic features that addresses the previous shortcomings. This method allows the user to input a set of genomic features of interest and receive an annotated ranked list of these features and their corresponding statistical significance, quantified by appropriately computed *p*- and *q*-values [to control the false discovery rate (FDR)]. The proposed approach evaluates, within a genomic feature of interest, DNA methylation discordance between a test and a reference phenotype, by quantifying the amount of information that the methylation state of a genomic feature contains about the phenotype, and by using this information to score the genomic feature. This is accomplished by articulating an appropriate hypothesis testing problem whose test statistic is derived using the mutual information between the methylation state and the phenotype. Importantly, this test statistic can be directly evaluated from WGBS data by computing JSD values within a given genomic feature using informME.

To address the confounding issue of feature length variability, we assume that the null probability density function (PDF) of the test statistic required for hypothesis testing depends on the length of the feature. We then approximate this PDF using the logit skewed Student’s *t* distribution (logitSST) with length-dependent parameters, which we estimate from replicate reference WGBS data using heteroscedastic regression. We verify the appropriateness of the logitSST model for estimating the PDF of the test statistic under the null hypothesis by performing goodness-of-fit and model selection analyses.

To illustrate the utility of the proposed method and its integration with informME, we reanalyzed previously available WGBS data obtained from healthy individuals and patients diagnosed with two different types of pediatric high-grade gliomas (pHGGs), a highly malignant form of brain tumor in children. Using these data, we ranked genes in terms of observed methylation discordance within their promoter regions and gene bodies, as well as within bivalent domains, whose role in the epigenetic regulation of gene expression is increasingly acknowledged in the literature [14]. Our results provide a clear demonstration of the importance and credibility of the proposed approach for linking genome-wide WGBS analysis results back to specific genomic features of interest and for appropriately ranking these features using their statistical significance. Our analysis shows that our method is capable of identifying genes that have been previously reported in the literature to be important in pHGGs, illustrates the importance of using multiple features (e.g., promoter regions and gene bodies) for ranking genes, demonstrates its seamless integration with informME, and establishes its importance as an exploratory tool in a WGBS analysis framework that can effectively identify important genomic regions and features for subsequent in-depth analysis.

We have coded the proposed method using R and have integrated it with informME. A fully documented GPLv3 licensed software implementation can be downloaded fromGitHub (https://github.com/GarrettJenkinson/informME).

## Methods

### Information-theoretic analysis of methylation

In a previous work [7, 8], we developed an information-theoretic approach to the modeling and analysis of WGBS data known as informME. This methodology performs methylation data analysis by partitioning the genome into non-overlapping regions and by estimating the probability mass function (PMF) of the methylation state within these regions genome-wide. Let us consider one of these genomic regions comprised of *N* CpG sites 1,2,…,*N*, which are indexed by their order of appearance along the genome. informME associates with the *n*-th CpG site a binary random variable *X*_*n*_ that takes values 0 or 1 if the site is unmethylated or methylated, respectively. It then characterizes the methylation state **X**=(*X*_1_,*X*_2_,…,*X*_*N*_) by a (usually) high-dimensional joint probability distribution Pr[ **X** = ***x***], which is modeled using the 1D Ising model of statistical physics estimated from available WGBS data using statistical inference. To summarize this high-dimensional probability distribution, informME performs methylation analysis by partitioning the genome into small analysis regions of 150bp each, which we refer to as genomic units (GUs). The methylation state within a GU with *L* CpG sites *ℓ* = 1,2,…,*L* is then characterized by the methylation level $M \!\,=\,\! \frac {1}{L} \sum _{\ell =1}^{L} X_{\ell }$, whose probability distribution Pr[*M* = *m*], *m* = 0,1/*L*,…,1, is computed from the estimated Ising distribution Pr[ **X** = ***x***]. In turn, this produces two statistical summaries of interest that we use to describe the statistical behavior of methylation within a GU: the mean methylation level (MML), given by $\mathrm {E}[M]=\!\!\!\!\!\sum _{m} m \Pr [\!M\!\!\,=\,\!\!m]$, and the normalized methylation entropy (NME), given by $h = -\left \{ \sum _{m} \Pr [M=m] \text {log}_{2} \Pr [M=m] \right \}\! / \text {log}_{2}(L+1)$.

### Mutual information and test statistic

In this paper, we are interested in statistically detecting DNA methylation discordances between a test and a reference phenotype within genomic features of interest, specified by their start and end coordinates along the genome. In particular, we seek to score genomic features in terms of the potential of their methylation states to distinguish between the two phenotypes. Genomic features of interest might include gene promoters, gene bodies, exons, introns, enhancers, bivalent domains, or any other genomic regions deemed to be important in a specific application.

A powerful way to proceed is to evaluate the dependance of the methylation state on the phenotype using the concept of mutual information [15]. In the following, we model the phenotype by a random variable *Q* that takes values 1 or 0, indicating a test or a reference phenotype, respectively. Moreover, we specify the methylation state of a genomic region by the *K*-dimensional random vector ***M***=(*M*_1_,*M*_2_,…,*M*_*K*_), where *M*_*k*_ is the methylation level of the *k*-th GU with data (i.e., with computed MML, NME and JSD values) that overlaps the region. We can then measure the dependance of ***M*** on *Q* using the average mutual information within the genomic region, given by

1 $$ \overline{I}(\boldsymbol{{M}};Q) = \frac{1}{K}\!\sum_{k=1}^{K} I(M_{k};Q),  $$

where

2 $$ \begin{aligned} I(M_{k};Q) \!\!= \!\! \sum_{q=0,1} \sum_{m_{k}} \Pr[M_{k}\,=\,m_{k},Q\,=\,q] \log_{2} \frac{\Pr[M_{k}=m_{k},Q=q]}{\Pr[M_{k}=m_{k}]\Pr[Q=q]}.  \end{aligned}  $$

In Eqs. (1) & (2), *I*(*M*_*k*_;*Q*) is the mutual information between *M*_*k*_ and *Q*, which tells us how much information the methylation state within the *k*-th GU carries about the phenotype and accounts for higher order relationships between the variables than simple correlations.

In the absence of any prior information about the phenotype, we can set Pr[*Q* = 1]= Pr[*Q* = 0]=1/2. In this case, we can show (Additional file 1: Section 1) that $\overline {I}(M;Q) = (1/K) \sum _{k=1}^{K} [\text {JSD}(k)]^{2}$, where JSD(*k*) is the Jensen Shannon distance (JSD) [16] between the conditional methylation PMFs Pr[*M*_*k*_ = *m*_*k*_ ∣ *Q* = 1] and Pr[ *M*_*k*_ = *m*_*k*_ ∣ *Q* = 0] of the test and reference phenotypes within the *k*-th GU, respectively. This result motivates us to statistically score epigenetic discordance within a genomic region using $1/K \sum _{k=1}^{K} \left [\text {JSD}(k)\right ]^{2}$ as the test statistic, since large values of this quantity indicate that DNA methylation within the region carries, on the average, significant information about its phenotypic state. However, for reasons we explain in Additional file 1: Section 2, we would like our test statistic to satisfy the triangle inequality *T*(*q*_1_,*q*_2_)+*T*(*q*_1_,*q*_3_)≥*T*(*q*_2_,*q*_3_), where *T*(*p*,*q*) is the test statistic used to distinguish between two phenotypes *p* and *q*. It turns out that this is not true for $1/K \sum _{k=1}^{K} \left [\text {JSD}(k)\right ]^{2}$. However, we can show that it is true for

3 $$ T = \sqrt{\frac{1}{K} \sum_{k=1}^{K} \left[\text{JSD}(k)\right]^{2}}  $$

(see Additional file 1: Section 2), which is the test statistic we use in this paper. Notably, *T* is a normalized test statistic that takes its minimum value 0 when the methylation state within the genomic region carries no information about the phenotypic state and its maximum value 1 when the methylation state is maximally informative about the phenotypic state (Additional file 1: Section 2). Moreover, *T* can be readily computed from available WGBS data, since the JSD can be calculated using informME [8].

### Scoring genomic features

Two important issues arise when using the test statistic *T* in Eq. (3) to score a genomic feature: scoring is subject to biological, statistical, and technical variability, whereas the test statistic depends on the number of GUs with data that overlap the genomic region associated with the feature, which is affected by its length as well as by the number of overlapping GUs with missing data. Although we can sufficiently address the first issue by collecting replicate reference data, by performing all possible reference/reference comparisons, and by properly including the results of such analysis in the test/reference hypothesis testing problem, the second issue is more complex. In this section, we propose a method that takes into account biological, statistical, and technical variability, as well as the “sizes” of the genomic features under consideration. We quantify the size of a genomic feature by *s*= log2*K*, where we introduce the logarithm to handle the large dynamic range of the number *K* of GUs with data that overlap the corresponding genomic region. In a given application, we may also wish to consider genomic features with sizes no smaller than a minimum size *s*_min_= log2(*K*_min_) to ensure that only features with sufficient length and/or data enter into the analysis.

We can simultaneously address the previous issues by computing the *null* PDF *f*_0_(*t*;*s*) of the test statistic *T* associated with a genomic region of size *s*, under the hypothesis that methylation discordance observed within the region is only due to biological, statistical, and technical variability. We can then compute the *p*-value, associated with an observation *t*_∗_ of *T* in a test/reference comparison for a genomic feature of size *s*, by $p(s)=\int _{t_{*}}^{1} f_{0}(t;s) dt$, and use this *p*-value to rank the genomic region based on evidence against the null hypothesis that the observed value *t* can be explained by normal biological, statistical, and technical variability.

In theory, we could use replicate reference WGBS samples to empirically estimate *f*_0_(*t*;*s*). However, for this estimation to be sufficiently accurate, it is required that a large number of reference replicate data must be available and a prohibitively large number of reference/reference comparisons must be performed, which is not feasible in practice. We address this problem by employing a recently developed method for heteroscedastic regression, which we discuss next.

For a size *s*, we assume that the null PDF *f*_0_(*t*;*s*) can be sufficiently approximated by a logit skewed Student’s *t* distribution (logitSST) *ϕ*(*t*;*θ*_*μ*_(*s*),*σ*(*s*),*ν*(*s*),*τ*(*s*)) with parameters *μ*, *σ*, *ν*, and *τ* that depend on *s*, in which case we set

4 $$ f_{0}(t;s) \simeq \widehat{f}_{0}(t;s) = \phi(t;\mu(s), \sigma(s),\nu(s),\tau(s)).  $$

The logitSST distribution is the PDF of the random variable *Y*=1/(1+*e*^−*X*^), −*∞*<*X*<*∞*, where *X* follows the skewed Student’s *t* distribution [17].

To compute $\widehat {f}_{0}(t;s)$ in Eq. (4), we need to estimate the four parameters *μ*(*s*), *σ*(*s*), *ν*(*s*) and *τ*(*s*) for any size *s* using observations of the test statistic *T* obtained from the reference samples. We perform this task by using the gamlss package of R [18], which assumes that *μ*(*s*), ln*σ*(*s*), ln*ν*(*s*), and ln*τ*(*s*)−2 are smooth functions of *s* [19] and estimates these functions from data using generalized additive regression models based on penalized splines [18, 20]. Smoothness ensures that the approximation $\widehat {f}_{0}(t;s)$ of the null PDF will be changing smoothly as the size *s* varies. In the “Results” section, we perform goodness-of-fit and model selection analyses to verify that the logitSST model provides an acceptable approximation to *f*_0_(*t*;*s*).

### Scoring genes in a single test/reference comparison

As an example of the method we propose for scoring genomic features, we rank genes based on their significance in exhibiting differential DNA methylation discordance in a test/reference sample. One way to do this is to rank genes based on methylation discordance within their promoters. Towards this goal, we perform hypothesis testing by employing the test statistic *T*_*p*_ calculated within promoter regions using Eq. (3), and score the significance of each gene using the computed *p*-value for that gene, where higher significance is associated with a lower *p*-value. We can also use a similar approach to rank genes based on methylation discordance within their bodies by employing the test statistic *T*_*b*_ calculated within gene bodies.

In addition to the previous rankings, we may obtain a more informative gene ranking if we could simultaneously test for methylation discordance within their promoters and bodies. In this case, and for each gene, we can test the null hypothesis that methylation discordance between a test and a reference WGBS sample observed within its promoter region and gene body is only associated with biological, statistical, or technical variability in the reference sample, against the alternative hypothesis that this discordance is due to other factors within at least one of the two genomic regions (promoter or gene body). We can perform this hypothesis testing using Fisher’s summary test statistic [13]

5 $$ T_{pb} = -2 \ln P_{p} - 2 \ln P_{b},  $$

where *P*_*p*_ and *P*_*b*_ are the *p*-values obtained by separately testing, using the test statistics *T*_*p*_ and *T*_*b*_, respectively methylation discordance within promoter regions or gene bodies.

If the two hypothesis testing problems were statistically independent, then *T*_*pb*_ would follow, under the combined null hypothesis, a $\chi ^{2}_{4}$ distribution with 4 degrees of freedom [13] from which a *p*-value for rejecting the combined null hypothesis could be readily obtained. However, due to the correlative nature of DNA methylation, the individual hypothesis tests may in general depend on each other, in which case, *T*_*pb*_ will not follow a $\chi ^{2}_{4}$ distribution.

To address this problem, we characterize the test statistic *T*_*pb*_ using its cumulative distribution function (CDF) *F*_*pb*_(*t*), which we empirically estimate by

6 $$ \widehat{F}_{pb}(t) = \frac{1}{N_{r}}\sum_{n=1}^{N_{r}} I\left[t_{n}\leq t\right],  $$

where *N*_*r*_ is the number of observations *t*_*n*_, *n*=1,2,…,*N*_*r*_, of the test statistic *T*_*pb*_ in the reference samples, and *I*[·] is the Iverson bracket, taking value 1 when its argument is true and 0 otherwise. In contrast to the theoretical $\chi ^{2}_{4}$ distribution, using $\widehat {F}_{pb}(t)$ will incorporate existing correlations into the problem and result in a more conservative and accurate statistical analysis than using the $\chi ^{2}_{4}$ distribution. In the “Results” section, we perform goodness-of-fit analysis and show that the empirical CDF $\widehat {F}_{pb}(t)$ provides a more appropriate characterization of the probability distribution of the test statistic *T*_*pb*_ than the theoretically derived $\chi ^{2}_{4}$ distribution.

As a consequence of the above, to rank genes based on observed methylation discordance within their promoters and bodies, we perform hypothesis testing using the *T*_*p*_ and *T*_*b*_ statistics to calculate (genome-wide) the *p*-values *P*_*p*_ and *P*_*b*_ for a given test/reference comparison, and compute the values of the test statistic *T*_*pb*_ using Eq. (5). For a given gene with observed test statistic value *t*_∗_ between the test and the reference samples, we use the estimated null CDF $\widehat {F}_{pb}(t)$ in Eq. (6) and approximately calculate the probability (*p*-value) $P_{pb} \simeq 1 - \widehat {F}_{pb}(t_{*})$ that, under the null hypothesis (of methylation discordance between a test and a reference sample observed within the gene’s promoter region and body being only associated with biological, statistical, and technical variability), the test statistic *T*_*pb*_ is at least as large as the observed value *t*_∗_. We then employ this *p*-value to score the gene and use these scores to rank genes in terms of the significance of their methylation discordance in the test sample, with higher significance being associated with a lower score.

### Scoring genes in multiple test/reference comparisons

We can also score a gene when multiple pairs of test/reference samples are available. To do so, we test the null hypothesis that epigenetic discordance observed within its promoter region and gene body in the test/reference comparisons is only associated with biological, statistical, or technical variability in the reference samples, against the alternative hypothesis that this discordance is due to other factors within at least one of the two genomic regions (promoter or gene body) in at least one of the test/reference samples. To address this problem, we use Fisher’s summary test statistic

7 $$ T_{\text{mult}} = -2 \sum_{n=1}^{N_{t}} \: \ln P^{(n)},   $$

where *N*_*t*_ is the number of available test/reference comparisons and *P*^(*n*)^ is the *p*-value obtained from the previous single comparison hypothesis testing problem (i.e., *P*_*p*_, *P*_*b*_, or *P*_*pb*_) applied on the *n*-th test/reference pair.

The individual hypothesis testing problems can be considered to be statistically independent, in which case, the test statistic *T*_mult_ follows a $\chi ^{2}_{2N_{t}}$ distribution with 2*N*_*t*_ degrees of freedom under the null hypothesis. From this distribution, we can compute a *p*-value for rejecting the null hypothesis, which we can use to score a gene’s significance in terms of its methylation discordance in the test samples and produce a list of ranked genes. Note however that there might be ties in the resulting list, due for example to numerical issues that do not allow calculation of the *p*-values with arbitrary precision. We break such ties to the extent possible by using another list of ranked genes produced for this purpose, which we generate by combining the rankings obtained from each single test/reference comparison using the method of rank products [21]. Finally, to evaluate the statistical significance of each ranking while controlling for the FDR, we compute *q*-values using the Benjamini-Hochberg procedure [22].

## Results

### WGBS data samples

We now demonstrate the applicability of the proposed ranking method by reanalyzing, using informME, previously available WGBS data (Additional file 2: Table S1) obtained from normal fetal brain tissue or primary patient-derived pHGG tumor samples. For test samples, we use pHGG WGBS data from [23], which includes 7 primary pHGG samples harboring the H3.3 K27M mutation [a mutation within the histone H3.3 gene *H3F3A* that results in substitution of lysine 27 on the amino-terminal tail of H3.3 with methionine (K27M)], and 6 primary pHGG samples without K27M mutations (H3.3-WT). For reference samples, we use data (four samples) from normal fetal cerebellum tissue [24]. Boxplots of genome-wide distributions of JSD values obtained from our WGBS data confirm the appropriateness of the reference samples for providing a quantitative assessment of normal biological, statistical, and technical variability in our analyses (Additional file 1: Figure S1).

### Goodness of fit

#### Estimation of null PDF of the *T* statistic

To demonstrate the appropriateness of the logitSST distribution for approximating the null PDF *f*_0_(*t*;*s*) of the test statistic *T* in Eq. (3), we considered a number of statistical models available in the gamlss R package and fitted each model to promoter region and gene body null *T* statistics obtained from six reference/reference comparisons. We then compared the results by employing two different model selection criteria, namely Akaike’s information criterion (AIC) and the Bayesian information criterion (BIC). We took the promoter region of a gene to be the genomic region covered by a 4-kb window centered at the gene’s TSS, and its gene body to be the genomic region between the gene’s TSS and its termination site that does not overlap with its promoter region. We obtained this information by using the R package TxDb.Hsapiens.UCSC.hg19.knownGene. Moreover, we considered non-inflated and inflated distribution models based on the beta, generalized beta type 1, logit normal, logit t-family, and logit skewed Student’s *t* distributions. The inflated models considered here include extra parameters to account for discrete probabilities of the test statistic taking its minimum and maximum values of 0 and 1 [18].

Ours results, summarized in Table 1, clearly show that the non-inflated logitSST model is superior under both criteria, in the sense that it produces the lowest AIC and BIC values, and this is true regardless of the type of genomic features considered (promoters or gene bodies). This model uses 25 degrees of freedom when fitted to promoter regions and 24 degrees of freedom when fitted to gene bodies. Note that a lower AIC means that the logitSST model is considered to be closer to the true model, whereas a lower BIC means that the logitSST model is considered more likely to be the true model.

**Table 1:** AIC and BIC values, computed using gamlss, for a number of non-inflated and inflated distribution models of the null probability density function of the *T* statistic, along with their (rounded) effective degrees of freedom (DF)

	Non-inflated Model			Inflated Model		
Model	AIC	BIC	DF	AIC	BIC	DF
Promoter						
BE	-486,026	-485,855	17	-486,229	-486,041	19
GB1	-488,175	-487,961	22	-488,408	-488,181	23
logitNO	-487,985	-487,895	9	-492,544	-492,384	16
logitTF	-493,963	-493,792	18	-493,944	-493,762	19
**logitSST**	**-494,260**	**-494,019**	**25**	-494,242	-493,992	26
Gene body						
BE	-482,633	-482,474	17	-482,608	-482,440	18
GB1	-484,822	-484,615	27	-484,798	-484,581	23
logitNO	-490,890	-490,730	17	-490,872	-490,702	18
logitTF	-495,777	-495,594	19	-495,751	-495,560	20
**logitSST**	**-498,362**	**-498,133**	**24**	-498,337	-498,099	25

The results were obtained by fitting each model to *T* statistic values within promoter regions and gene bodies obtained from all six reference/reference comparisons. Entries in bold highligth the proposed model, which is shown here to produce the best AIC and BIC scores

BE: beta distribution; GB1: generalized beta type 1 distribution; logitNO: logit normal distribution; logitTF: logit t-family distribution; logitSST: logit skewed Student’s *t* distribution

In addition to the previous important result, we can also demonstrate that logitSST produces a high-quality fit to the null distribution of the *T* statistic data computed from the reference samples. Note that the size of gene bodies varies substantially more than the size of promoter regions. This is due to the fact that the size of a genomic feature is influenced by its actual length in bp’s, which we choose to be fixed at 4-kb for the case of promoter regions. For this reason, we focus our discussion here on gene bodies. A similar approach applies for the case of promoter regions (see Additional file 1 for results pertaining to promoters).

To compute the approximation $\widehat {f}_{0}(t;s)$ of the true null PDF *f*_0_(*t*;*s*) of the *T* statistic within gene bodies, we applied informME on the four normal fetal cerebellum tissue samples, computed the JSD values in all six comparisons genome-wide, and calculated the *T* statistic values within all gene bodies via Eq. (3). To ensure that only features with sufficient length and/or data enter into our analysis, we considered only gene bodies with sizes *s*≥*s*_min_= log2(10) (i.e., we considered only bodies that overlap at least 10 GUs with data). This resulted in 104, 694 paired observations (*t*_*k*_,*s*_*k*_) of null *T* statistic values *t*_*k*_ and gene body sizes *s*_*k*_, which we passed to gamlss to produce $\widehat {f}_{0}(t;s)$.

**Fig. 1:**
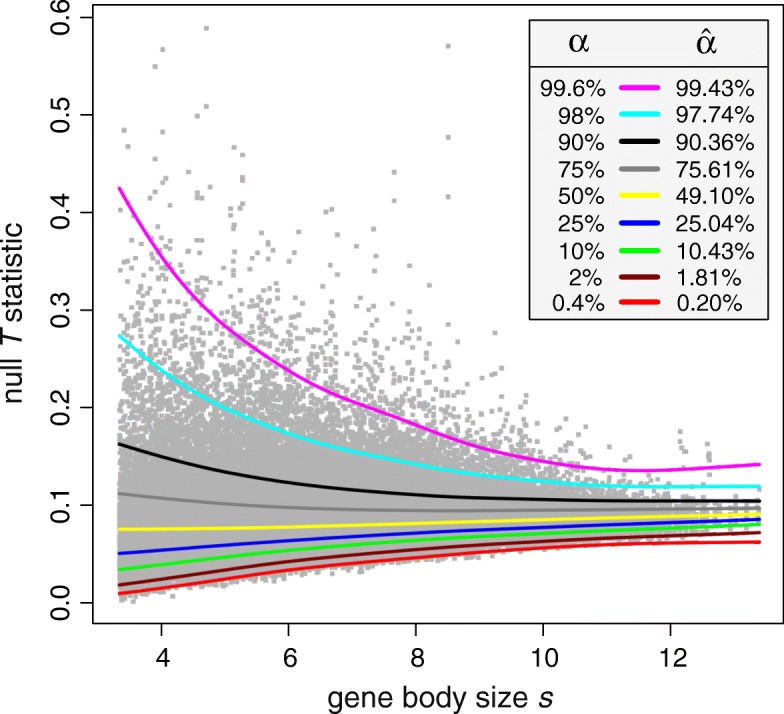
*α*-centile curves, calculated for different values of *α* from the estimated logitSST-based null PDF $\widehat {f}_{0}(t;s)$ within gene bodies, drawn over a scatter plot of 104, 694 observed pairs (*t*_*k*_,*s*_*k*_) of null *T* statistic values *t*_*k*_ and gene body sizes *s*_*k*_. The percentage $\widehat {\alpha }$ of empirically observed data points that fall below a centile curve agrees well with the corresponding *α* value, indicating that $\widehat {f}_{0}(t;s)$ is consistent with the data

In Fig. 1, we depict a scatter plot of pairs of (*t*_*k*_,*s*_*k*_) values for the case of gene bodies, together with *α*-centile curves computed from the estimated logitSST-based null PDF $\widehat {f}_{0}(t;s)$ using different values of *α* (see Additional file 1: Figure S2 for results pertaining to promoter regions). An *α*-centile curve indicates that *α%* of the data points are below the curve. The results demonstrate that the estimated centile curves match the data very well. This is due to the fact that the percentage $\widehat {\alpha }$ of the empirically observed data points below a given estimated centile curve is close to its centile value *α*, indicating that the estimated null PDF $\widehat {f}_{0}(t;s)$ is consistent with the data. Note also that the data depicted in Fig. 1 demonstrate clear heteroscedasticity, indicating that the null PDF *f*_0_(*t*;*s*) depends on the gene body size *s*, as expected.

We further assessed the goodness of fit of the estimated PDF $\widehat {f}_{0}(t;s)$ using quantile residuals [25], as we explain next. Let *F*_0_(*t*;*s*) and $\widehat {f}_{0}(t;s)$ be the CDFs respectively associated with the true (but unknown) null PDF *f*_0_(*t*;*s*) and the logitSST-based estimated null PDF $\widehat {f}_{0}(t;s)$. Note that, for any size *s*, *U*=*F*_0_(*T*;*s*) is a random variable that is uniformly distributed over the unit interval [0,1] regardless of the particular PDF of the *T* statistic. This implies that *G*=*Φ*^−1^(*U*)=*Φ*^−1^(*F*_0_(*T*;*s*)), where *Φ* is the CDF of the standard normal distribution, is a zero-mean Gaussian random variable with unit standard deviation. We therefore expect that, when the logitSST-based estimated null PDF $\widehat {f}_{0}(t;s)$ is a good approximation of the true null PDF *f*_0_(*t*;*s*), the estimated quantile residuals, computed by $g_{k} = \Phi ^{-1}(\widehat {F}_{0}(t_{k};s_{k}))$ will be samples drawn from the standard normal PDF, where *t*_1_≤*t*_2_≤…. If this turns-out to be true, then we can claim that the estimated null PDF provides a good fit of the true null PDF.

**Fig. 2:**
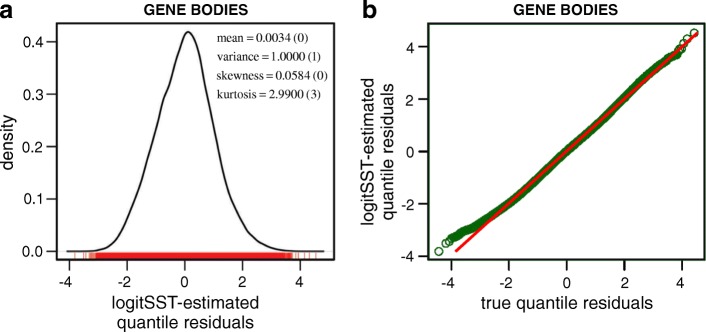
Quantile residual analysis of the logitSST-estimated null PDF of the *T* statistic in the case of gene bodies. **a** The kernel density approximation of the distribution of the logitSST-estimated quantile residuals (bottom red marks) demonstrates close agreement with standard normality. **b** The Q-Q plot (green marks) of the logitSST-estimated quantile residuals against the corresponding true quantile residuals is very close to the diagonal (red) line, suggesting a close agreement of the logitSST-estimated null PDF of the *T* statistic to its true distribution

In Fig. 2a, we depict a kernel density approximation of the logitSST-estimated quantile residuals (shown in red at the bottom of the figure), obtained for the case of gene bodies using the reference samples [see Additional file 1: Figure S3a for the case of promoter regions]. Moreover, we provide values of four measures of location and shape of the approximated PDF, with the values in parentheses corresponding to the standard normal distribution. This result shows that the logitSST-estimated quantile residuals follow a probability distribution that is very close to being standard normal. We also depict in Fig. 2b a quantile-quantile (Q-Q) plot (green marks) of the logitSST-estimated quantile residuals for the case of gene bodies against the corresponding true quantile residuals [see Additional file 1: Figure S3b for the case of promoter regions]. The fact that the Q-Q plot is very close to the diagonal (red) line, is another indication of standard normality of the logitSST-estimated quantile residuals obtained from the reference samples.

#### Estimation of null CDF of the *T*_*pb*_ statistic

As we discussed earlier, scoring genes in a single test/reference comparison requires evaluation of the null probability distribution of the test statistic *T*_*pb*_ in Eq. (5). Due to the correlative nature of methylation, *T*_*pb*_ may not follow a $\chi ^{2}_{4}$ distribution, as theoretically expected by Fisher’s method. For this reason, we chose to approximate the true null CDF of the test statistic *T*_*pb*_ using the empirical estimate $\widehat {F}_{pb}(t)$, given by Eq. (6).

**Fig. 3:**
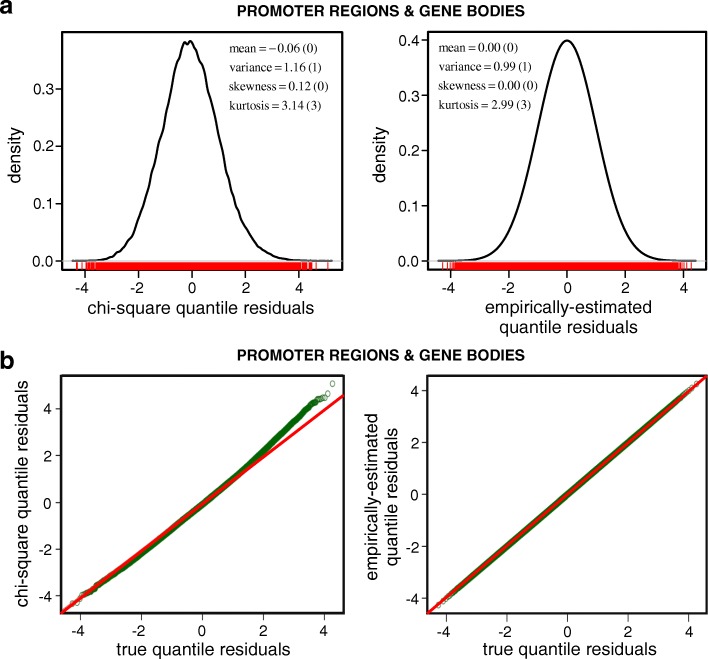
Quantile residual analysis of the empirically-estimated null CDF of the *T*_*pb*_ statistic versus the null CDF associated with the $\chi ^{2}_{4}$ distribution, theoretically suggested by Fisher’s method. **a** The kernel density approximations of the distributions of the $\chi ^{2}_{4}$ (left) and the empirically-estimated (right) quantile residuals demonstrate closer agreement of the latter with standard normality than the former. **b** The Q-Q plot (green marks) of the $\chi ^{2}_{4}$ (left) and the empirically-estimated (right) quantile residuals against the corresponding true quantile residuals corroborate the result in (**a**)

To show the superiority of empirically approximating the null CDF of *T*_*pb*_, we again performed quantile residual analysis. In Fig. 3a, we depict kernel density approximations of the quantile residuals obtained by using the null CDF associated with the $\chi ^{2}_{4}$ distribution (left), as well as the empirical null CDF (right), computed by using Eq. (6). The values of the location and shape parameters associated with these densities demonstrate that the empirical CDF provides a better approximation to the true distribution, since the computed values in this case are closer to the ones that correspond to the standard normal distribution (values in parentheses). This is also corroborated by the Q-Q plots depicted in Fig. 3b. In the Q-Q plot that corresponds to the $\chi ^{2}_{4}$ distribution (left), large quantile residuals exhibit noticeable deviation from the true residuals. However, the Q-Q plot corresponding to the empirical distribution (right) shows excellent match, indicating that this distribution is very close to the true distribution.

### Gene ranking in pHGGs

In a previous paper [8], we introduced a method for identifying genes that exhibit significant epigenetic discordance within their promoters in a test/reference comparison. However, this method will miss genes that show significant epigenetic discordance only within their gene bodies. To find such genes, we may attempt to use a commonly applied heuristic that identifies genes overlapping differentially methylated regions (DMRs) of the genome determined by an effective DMR finder, such as the JSD-based DMR (jsDMR) detector of informME [8]. Nevertheless, results obtained by this method are confounded by gene length, which is problematic from a statistical perspective. More importantly, there is a fundamental issue with this strategy when analyzing cancer data characterized by profound changes in DNA methylation, such as the K27M mutant samples we consider in this paper, since detected DMRs may cover most of the genome. In this case, we will not be able to differentiate between most genes, since DMR overlap will be exceedingly common. Moreover, genes will tend to be completely covered by large DMRs, implying that more detailed heuristics, such as scoring genes based on the percentage of their overlap with DMRs, will fail in this case.

To demonstrate these issues, we processed a K27M mutant sample using informME and applied the jsDMR finder. In Additional file 2: Table S2, we list all genes whose promoters or bodies overlap a jsDMR, as well as the location of the corresponding jsDMR. Nearly all genes overlap a jsDMR and, therefore, this “target” list of genes is of little use when attempting to identify important genes. Not surprisingly, using this list for quantitative assessment by means of gene ontology (GO) enrichment analysis [26] or gene set enrichment analysis (GSEA) [27], produces no significant results, since there is no opportunity for enrichment when nearly every gene is included in the target list.

To properly analyze the pHGG data, we applied the gene ranking method proposed in this paper to find genes exhibiting significant DNA methylation discordance within their promoter regions or gene bodies. We obtained results by comparing the H3.3-WT and K27M mutant pHGG samples to the normal fetal brain samples, which we summarize in Additional file 2: Tables S3 and S4, respectively.

As expected, a large number of genes demonstrate statistically significant methylation discordance (*q*-value ≤0.05) in the combined promoter/body lists (PB lists). Crucially, however, our method retains the ability to rank the genes and find those that are most differentially methylated. Many of the top genes in the PB lists, such as *KHDRBS2*, *PCDHGA2*, *PCDHGA3*, *RALYL*, *SLC25A21*, and *THSD7B*, for the case of H3.3-WT, as well as *EPHA3*, *EPHA6*, *ESR1*, *HBE1*, *HLX*, and *MTUS2*, for the case of K27M mutant, are not highly ranked in terms of their promoter region alone. As a consequence, their importance could be missed if only the promoter region list (PR list) is used. These genes exhibit profound methylation discordance within their bodies and, for this reason, most are placed at the top of the gene body list (GB list). Notably, *KHDRBS2*, *RALYL*, *SLC25A21*, *THSD7B*, *EPHA3*, *ESR1*, *EPHA6*, *HBE1*, and *HLX* have been implicated in brain tumors [28–39], whereas *PCDHGA2*, *PCDHGA3*, and *MTUS2* have known relationships to other types of cancer [40, 41].

In addition to the above, by further investigating the PB list of ranked genes associated with H3.3-WT (Additional file 2: Table S3), we observed that homeobox (HOX) genes, which are important in determining cell fate and identity during embryonic development and have been implicated in many cancers, rank particularly high in that list. Notably, it was recently suggested that *HOXB3*, a HOX gene ranked at the top of the PB list (although it is ranked 72 in the PR list and 45 in the GB list), promotes tumor cell proliferation and invasion in glioblastoma [42]. Moreover, *HOXA9* has been implicated as an oncogene in glioblastoma and its aberrant expression seems to be independently predictive of shorter survival rates [43]. In addition, an expression signature dominated by HOX genes has also emerged as a predictor for poor survival in patients treated with concomitant chemo-radiotherapy [44].

To further assess the biological significance of our gene rankings, we performed GO analysis using the PB list of genes ranked based on their discordance within their promoter regions and gene bodies, which we obtained from Additional file 2: Table S3 (for H3.3-WT) and Additional file 2: Table S4 (for the K27M mutant). We summarize the results in Additional file 2: Tables S5 and S6. Notably, GO analysis respectively identified 1293 and 232 significantly enriched GO process categories in the case of H3.3-WT and K27M mutant, indicating that our rankings are highly enriched near the top with genes of known biological significance. The GO results include several biological processes that play important roles in cancer initiation and progression, such as signaling, transcription regulation, cell communication, differentiation, commitment, morphogenesis, migration, and motility, as well as processes involved specifically in neuron development, differentiation, commitment, proliferation, and migration.

To bolster the biological relevance of the top genes in our lists, we also performed GSEA by using the top 500 significant genes from each ranked PB list and by computing overlaps with gene sets in the Molecular Signatures Database (MSigDB). We summarize these results in Additional file 2: Tables S7 and S8 for the case of H3.3-WT and K27M mutant, respectively. GSEA produced enrichments with gene sets related to stemness, as well as with genes known to exhibit DNA methylation and expression discordance in various cancers. In addition, GSEA showed a striking enrichment of genes whose promoters are bound by two functional enzymatic components (EED and SUZ12) of the Polycomb repressive complex 2 (PRC2), which promotes chromatin compaction by establishing, through another enzymatic component (EZH2), dimethylated H3K27 (H3K27me2) and trimethylated H3K27 (H3K27me3) marks [45]. This implies that, in pHGG, PRC2 targets genes that exhibit significant DNA methylation discordance.

The previous observation is not surprising, considering evidence that H3K27me3 and DNA methylation are compatible throughout most of the genome [46]. Notably, it has been suggested in [23] that the K27M mutation acts as a dominant negative inhibitor of H3K27 di- and trimethylation, due to an aberrant recruitment of the PRC2 complex to K27M mutant H3.3 and reduction of PRC2 activity through enzymatic inhibition of EZH2, and that this behavior is accompanied by hypomethylation, which is clearly seen in our K27M mutant samples (see Additional file 1: Figure S1). As a consequence, it has been suggested in [23] that inhibition of PRC2 activity and DNA hypomethylation may provide a mechanism of an altered gene expression program that drives tumor progression in K27M mutant cells. However, our finding that PRC2 also targets genes that exhibit significant DNA methylation discordance in H3.3-WT pHGG and the fact that this type of tumor exhibits hypomethylation as well (see Additional file 1: Figure S1), raises the possibility that a similar mechanism involving DNA hypomethylation at PRC2-regulated sites may also explain aberrant gene expression in H3.3-WT cells that leads to tumorigenesis.

In addition to the above, our observation that PRC2-regulated genes play a central role in both H3.3-WT and K27M mutant tumors is particularly intriguing, considering our previous suggestion that the PRC2 complex may be an important regulator of epigenetic stochasticity [7]. In the present framework, this is supported by the fact that both H3.3-WT and K27M mutant samples are globally characterized by gains in methylation entropy in most of our pHGG data (see Additional file 1: Figure S1).

**Fig. 4:**
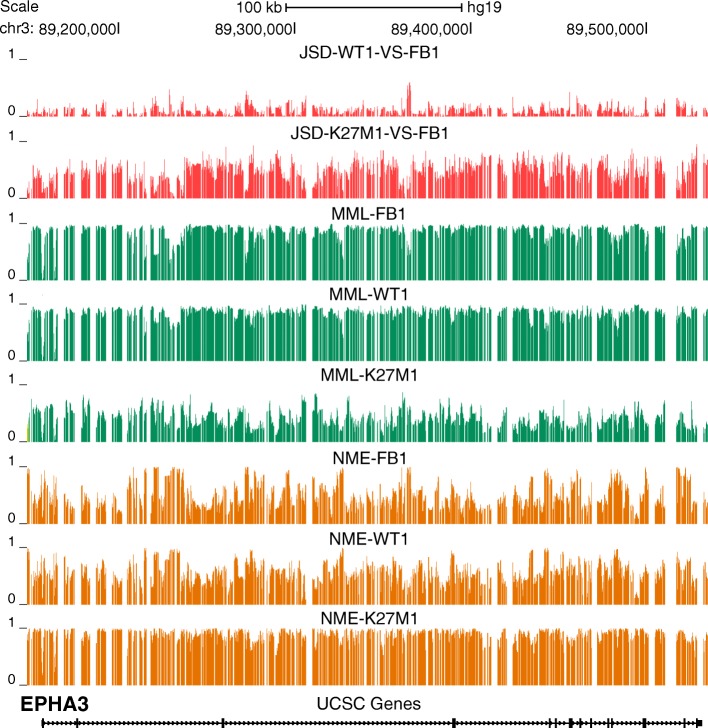
UCSC genome browser images of JSD, MML, and NME tracks within the genomic region [chr3: 89,145,180–89,536,200] that contains *EPHA3*, obtained by informME in the WT1 vs. FB1 and K27M1 vs. FB1 comparisons. The JSD tracks indicate small differences in DNA methylation when comparing WT1 to FB1, but large differences when comparing K27M1 to FB1. This is associated with widespread hypomethylation in K27M1 (MML tracks) and a gain in methylation entropy (NME tracks) close to its maximum value

After identifying a gene of interest using our ranked lists, we can employ informME to further investigate properties of the methylation state within its promoter region and gene body in a test/reference comparison. In Fig. 4, we depict the JSDs, MMLs, and NMEs, computed by informME when comparing an H3.3-WT and a K27M mutant sample to a normal fetal brain sample, within a genomic region that contains the Ephrin type-A receptor 3 (*EPHA3*) gene. This gene ranks at the top of the PB list in the K27M sample (Additional file 2: Table S4), despite the fact that it is ranked 1874 in the PR list. This is due to the fact that *EPHA3* exhibits profound methylation discordance within its gene body (ranked at the top of the corresponding GB list). Interestingly, *EPHA3* does not rank highly when using the H3.3-WT samples (2965 in the PB list, 8976 in the PR list, and 1326 in the GB list; see Additional file 2: Table S3). Indeed, the JSD tracks depicted in Fig. 4 indicate small differences in DNA methylation when comparing the H3.3-WT sample to the fetal brain sample, but large differences within the gene body of *EPHA3* in the case of the K27M sample. In agreement with our previous discussion, this discordance is associated with appreciable hypomethylation, as well as with gain in entropy that approaches its maximum value, indicating a highly disordered methylation state over *EPHA3*. In Additional file 1: Figure S4, we demonstrate that this behavior is consistent across all samples.

The previous finding about *EPHA3* is of potential clinical significance, since it indicates that its methylation state is distinctly regulated in pHGG tumors harboring the K27M mutation. Importantly, *EPHA3* has been identified as a therapeutic target in glioblastomas using small-molecule inhibitors or targeted antibodies [33, 47]. However, our results provide a specific hypothesis regarding *EPHA3* targeting in pHGG: tumors harboring the H3.3 K27M mutation have a markedly dysregulated *EPHA3* epigenetic signature while H3.3-WT pHGGs do not, suggesting that *EPHA3* therapeutic targeting may be more effective in patients with the H3.3 K27M histone mutation. In agreement with this hypothesis, a recent paper employed super-enhancer profiling of patient-derived diffuse intrinsic pontine glioma (DIPG) cell lines to demonstrate that Ephrin signaling is crucial for DIPG invasiveness, which mostly exhibits the H3.3 K27M mutation [48]. Therefore, *EPHA3* warrants further investigation as a therapeutic target in pHGG, while taking into account the K27M mutational status of the underlying tumor, which might enable new precision medicine efforts for this disease cohort.

### Ranking bivalent domains in pHGGs

The results obtained by GSEA applied in H3.3-WT and K27M mutant pHGG (Additional file 1: Tables S7 and S8) revealed enrichment for genes in neural progenitor cells (NPCs) whose promoters bear the repressing H3K4 trimethylation mark (H3K4me3), as well as the activating histone H3K27 trimethylation mark (H3K27me3), which are distinctive histone modifications within bivalent domains [14]. Therefore, and as an additional example of the utility of our method for ranking genomic features, we sought to rank bivalent domains. The presence of bivalent domains within gene promoters and enhancers keeps the expression level of these “bivalent” genes at low levels, with the genes poised for rapid activation upon availability of suitable cues.

To determine bivalent domains, we used the genomic regions labeled “TssBiv” or “EnhBiv” under the ENCODE accession ENCSR567BIT. To ensure that only bivalent domains with sufficient length and/or data enter into the analysis, we considered only those with sizes *s*≥*s*_min_= log2(5) (i.e., we considered only bivalent domains overlapping at least 5 GUs with data). This resulted in 7446 paired observations (*t*_*k*_,*s*_*k*_) of null *T* statistic values *t*_*k*_ and bivalent domain sizes *s*_*k*_, which were passed to gamlss to produce $\widehat {f}_{0}(t;s)$. The goodness-of-fit results depicted in Additional file 1: Figures S5 and S6 are similar to the ones obtained for promoter regions and gene bodies and show that the logitSST-estimated null PDF $\widehat {f}_{0}(t;s)$ provides a good approximation of the true null PDF of the *T* statistic within bivalent domains as well.

In Additional file 2: Table S9, we provide a ranking of bivalent domains produced by our method when comparing the H3.3-WT pHGG samples to a fetal brain sample, as well as the genes located nearest to these domains. From this ranking, we identified many bivalent domains with significant discordance in DNA methylation, with the most highly ranked domains being associated, for example, with *ZNF467*, *PCDH8*, *ISLR2*, *HLX*, *NR2E1*, *WT1*, *AGAP2*, *TGFB1I1*, *LRFN1*, *KLF4*, and *SOX10*, which have been previously implicated in brain tumors [39, 49–58]. We obtained similar results when comparing the K27M mutant pHGG samples to the same fetal brain sample (Additional file 2: Table S10). In this case, however, some genes that were located lower in the previous list, such as *ATXN10*, *CLDN5*, *EBF4*, and *SOX9*, were found in the top of the list. These genes have also been implicated in brain tumors [59–62].

The previous results indicate that using our methodology to rank bivalent domains in test/reference studies can be quite useful for identifying important bivalent genes in a test/reference comparison for further experimental analysis and validation. In conjunction with informME, ranking bivalent domains and genes can also help to increase our understanding regarding their role in a particular disease, such as pHGGs. For example, when comparing the H3.3-WT samples to the normal fetal brain samples, a bivalent domain located at [chr22: 38,379,200–38,389,200] and associated with *SOX10* is ranked much higher than a bivalent domain located at [chr17: 70,112,800–70,114,000] and associated with *SOX9*, while the opposite is true when comparing the K27M mutant and normal fetal brain samples (see Additional file 2: Tables S9 and S10). Since these genes have been implicated in gliomas [58, 62–65], we sought to further investigate these differences using informME.

**Fig. 5:**
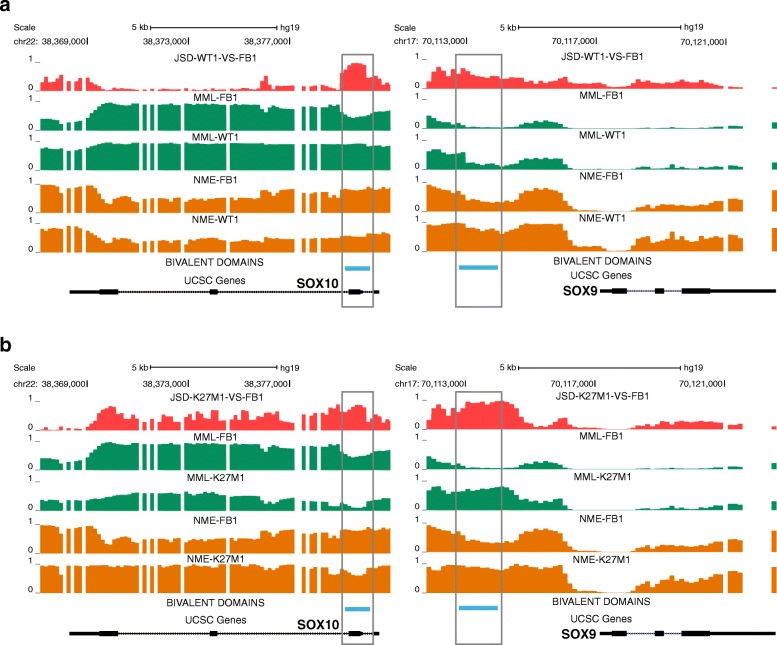
UCSC genome browser images of JSD, MML, and NME tracks obtained by informME within genomic regions [chr22: 38,367,000–38,381,000] and [chr17: 70,111,800–70,122,800] containing *SOX10* (left) and *SOX9* (right). **a** An H3.3-WT vs. normal fetal brain comparison. **b** A K27M mutant vs. normal fetal brain comparison

In Fig. 5a, we depict the JSDs, MMLs, and NMEs computed by informME when comparing an H3.3-WT sample to a normal fetal brain sample within regions that contain *SOX10* (left) and *SOX9* (right). The bivalent domain associated with *SOX10*, which partially overlaps its promoter region, exhibits strong DNA methylation discordance, as indicated by the large JSD values over this domain. This is associated with hypermethylation in the H3.3-WT sample accompanied by substantial loss of methylation entropy. Interestingly, hypermethylation of the SOX10 promoter was found to be associated with shorter survival rates in glioblastoma [58]. On the other hand, the bivalent domain associated with *SOX9* shows smaller DNA methylation discordance than the bivalent domain in *SOX10*, which can be associated with moderate hypermethylation in the H3.3-WT sample accompanied by an appreciable gain in methylation entropy.

Interestingly, *SOX9* and *SOX10* exhibit a different behavior when comparing K27M mutant pHGG to normal fetal brain. Figure 5b shows that the bivalent domain associated with *SOX10* (left) exhibits again strong DNA methylation discordance. However, this discordance is now associated with appreciable hypomethylation in the K27M mutant sample (as opposed to hypermethylation in the H3.3-WT case), accompanied by a moderate loss of methylation entropy. On the other hand, the bivalent domain associated with *SOX9* shows strong methylation discordance (as opposed to smaller discordance in the H3.3-WT sample), which is now associated with appreciable hypermethylation in the K27M mutant sample (as compared to the small hypermethylation in the H3.3-WT sample), accompanied by a substantial gain in methylation entropy.

The behavior of *SOX10* (but not of *SOX9*) within its associated bivalent domain in the K27M mutant is in agreement with the previously discussed finding suggesting that the K27M mutant is subject to extensive hypomethylation. This observation, together with the fact that K27M mutant pHGG is characterized by a global reduction in the repressive H3K27me3 marks, raises the possibility that expression of *SOX10* is activated in the K27M mutant, a hypothesis that has been recently validated in [66].

## Discussion

DNA methylation and its impact on cellular function has become a major focus of biomedical research. This is due to its role as a fundamental epigenetic mechanism in embryonic development and regulation of gene expression, as well as for being an important mediator between environmental risk factors and human diseases [2, 3, 67]. For this reason, several technologies have been developed to provide high throughput measurements of the DNA methylation state genome-wide, with WGBS being currently the best method. Computational analysis of methylation data obtained by WGBS allows extraction of epigenetic information that can be used, in conjunction with other biological data, to better understand the role of epigenetic regulation in health and disease [68].

WGBS data analyzed by software packages, such as informME [7, 8], present biologists with a large volume of epigenetic information that cannot be possibly reviewed and processed in a reasonable time, which poses a serious challenge when working with this type of data. It is therefore critical that, for efficient analysis, we develop computational tools that can direct attention to specific locations in the genome exhibiting statistically significant methylation discordances in test/reference comparisons. While the common practice of identifying DMRs can be helpful in this respect, they can also be difficult to interpret and analyze, especially in cases of widespread epigenomic changes, such as those typically seen in cancer.

From a statistical perspective, a more focused analysis within a set of genomic features of interest can ease some of the previous difficulties and provide higher statistical power. However, as we discussed earlier, methylation analysis is in general hampered by the presence of biological, statistical, and technical variability in the data, whereas a focused approach to this analysis is confounded by the variable length of most genomic features of interest as well as by missing data.

By using basic concepts from information theory and statistics, we have developed in this paper a new computational tool for methylation data analysis that effectively addresses the previous issues in a precise manner. This method passes a set of genomic features of interest to a rigorous statistical machinery that analyzes WGBS data obtained from a test/reference study and returns a list of ranked features together with corresponding *p*- and *q*-values.

An important practical question here is how to use our ranked lists for downstream WGBS data analysis. Our results provide methodological guidelines for the case of genes. We can begin with a manual investigation of the highest-ranking genes, which can provide familiarity with the results and leverage biological expertise. We can start at the top and move down the list gene-by-gene, allowing ourselves to adaptively decide how many genes we can afford to analyze using this labor-intensive strategy. However, we can also follow a more automated and unbiased strategy, for example using GO enrichment analysis based on a complete ranked list without having to specify an arbitrary threshold. In general, this should be considered a more preferable use of our ranked lists when comparing with methodologies that require separation of a given ranked list into two unranked categories of “target” and “background” genes. However, we did demonstrate in this paper with our GSEA results that using lists of target/background genes can still produce meaningful results and insights into the data, even when the decision on how many genes to include in the target set is challenging. Clearly, when a small or moderate number of genes are associated with significant *q*-values, these genes can be used to form a target set, but in the case of widespread epigenetic disruption some judgement and experience is required when selecting the number of genes that should be included in the target set. As a guideline, we have generally found that using around 500 genes in a target set strikes a good balance for GSEA analysis. If the target set is too large, there is too little power to detect enrichment, whereas if the list is made too small, then there may be important highly ranked genes (and thus enrichment categories) that will be missed by the analysis.

Finally, the data employed in this paper show that using logitSST regression for approximating the null PDF of our test statistic is superior to a number of alternative regression methods considered. Although we have also found this to be true in other WGBS studies, logitSST may not be the best regression method in general. To deal with the possibility that another regression method may be more preferable in a given study, a user may first consider a set of candidate regression methods, perform the type of “goodness of fit” and model selection analyses reported in this paper, and then replace logitSST with a better fitting model, if necessary.

## Conclusions

In this paper, we presented a rigorous approach for computing statistical significance of methylation discordance within genomic features from WGBS data. Our approach uses mutual information, a powerful information-theoretic tool for evaluating dependance of the methylation state on the phenotype, in conjunction with a well-articulated hypothesis testing problem and logitSST regression for estimating the null distribution, to score genomic features in terms of observed methylation discordance in differential studies. We showed that the test statistic associated with this hypothesis testing problem can be evaluated by computing, using informME [7, 8], the JSD between probability distributions of the methylation state within a test and a reference sample. We also suggested effective ways for implementing hypothesis testing in a way that takes into account biological, statistical, and technical variability, as well as variability in feature length and missing data in single-sample and multiple-sample comparisons. This was accomplished by estimating, from available reference data, the null distribution of our test statistic via a novel application of heteroscedastic regression based on generalized additive regression models using penalized splines.

By reanalyzing previously published pHGG data using the proposed method, we obtained results that add credibility to our scheme when ranking genes in terms of their promoters, bodies, or bivalent domains, since these results clearly demonstrate the potential of the method for identifying genes that have been previously reported in the literature to be crucial in glioblastomas. Moreover, our analysis shows that considering only methylation discordance within promoter regions of genes in a test/reference comparison may not be sufficient for identifying key genes, since methylation differences found within gene bodies and bivalent domains could be critical as well. We therefore believe that our approach can greatly facilitate the exploratory phases of clinically and biologically relevance methylation studies.

We should finally note that the idea of using regression models to build null distributions of test statistics from reference samples while accounting for confounding variables is quite general and can be applied to other problems of computational genomics. In addition to allowing a bioinformatician to handle confounding variables, such as feature length, our approach can naturally handle biological, statistical, and technical sources of variability, which are often overlooked by statistical tests based on theoretical/asymptotic sampling distributions. As such, we believe that the general statistical approach introduced in this paper can find widespread use for analyzing other types of whole-genome sequencing data, such as data obtained using chromatin immunoprecipitation sequencing (ChIP-seq) technologies.

## Additional files

Additional file 1 Supplementary material. This file contains additional method descriptions and supplementary figures. (PDF 3994 kb)

Additional file 2 Supplementary tables. This file contains supplementary tables summarizing our results. (XLSX 17,856 kb)

## Abbreviations

AIC: Akaike’s information criterion
BIC: Bayesian information criterion
CDF: Cummulative distribution function
DMR: Differentially methylated region
FDR: False discovery rate
GB: gene body
GO: Gene ontology
GSEA: Gene set enrichment analysis
GU: Genomic unit
informME: Information-theoretic analysis of methylation
JSD: Jensen-Shannon distance
jsDMR: Jensen-Shannon distance based differentially methylation region
logitSST: Logit skewed Student’s *t* distribution
MML: Mean methylation level
MSigDB: Molecular signatures database
NME: Normalized methylation entropy
NPC: Neural progenitor cell
PB: Promoter/body
PDF: Probability density function
PMF: Probability mass function
pHGG: Pediatric high-grade glioma
PR: Promoter region
TSS: Transcription start site
WGBS: Whole genome bisulfite sequencing
