# Supplementary material for: Ranking genomic features using an information-theoretic measure of epigenetic discordance
Source: BMC Bioinformatics. 2019 Apr 8;20:175. doi: 10.1186/s12859-019-2777-6 (PMC6454630; doi:10.1186/s12859-019-2777-6)
Supplement: Supplementary file 1 — Supplementary material. This file contains additional method descriptions and supplementary figures. (PDF 3994 kb) [file 12859_2019_2777_MOESM1_ESM.pdf]

SUPPLEMENTARY MATERIAL

Ranking genomic features using an information-theoretic measure of epigenetic discordance

G. Jenkinson, J. Abante, M. A. Koldobskiy, A. P. Feinberg, and J. Goutsias

1 Average mutual information and JSD

From Eqs. (1) and (2) of the Main paper, we have that

$$\begin{aligned}\bar{I}(\boldsymbol{M};Q) &= \frac{1}{K}\sum_{k=1}^{K}\sum_{q=0,1}\sum_{m_k}\Pr[M_k=m_k,Q=q]\,\log_2\frac{\Pr[M_k=m_k,Q=q]}{\Pr[M_k=m_k]\Pr[Q=q]}\\ &= \frac{1}{K}\sum_{k=1}^{K}\sum_{q=0,1}\Pr[Q=q]\sum_{m_k}\Pr[M_k=m_k\mid Q=q]\,\log_2\Pr[M_k=m_k\mid Q=q]\\ &\quad -\frac{1}{K}\sum_{k=1}^{K}\sum_{m_k}\Pr[M_k=m_k]\,\log_2\Pr[M_k=m_k].\end{aligned}$$

Then, by setting $\Pr[Q=0]=\Pr[Q=1]=1/2$ and by using the fact that

$$\begin{aligned}\Pr[M_k=m_k] &= \sum_{q=0,1}\Pr[M_k=m_k,Q=q]\\ &= \sum_{q=0,1}\Pr[M_k=m_k\mid Q=q]\Pr[Q=q]\\ &= \frac{1}{2}\sum_{q=0,1}\Pr[M_k=m_k\mid Q=q],\end{aligned}$$

we obtain

$$\begin{aligned}\bar{I}(\boldsymbol{M};Q) &= \frac{1}{2K}\sum_{k=1}^{K}\sum_{q=0,1}\sum_{m_k}\Pr[M_k=m_k\mid Q=q]\,\log_2\Pr[M_k=m_k\mid Q=q]\\ &\quad -\frac{1}{K}\sum_{k=1}^{K}\sum_{m_k}\Pr[M_k=m_k]\,\log_2\Pr[M_k=m_k]\\ &= \frac{1}{K}\sum_{k=1}^{K}[\mathrm{JSD}(k)]^2,\end{aligned}$$

where

$$\mathrm{JSD}(k)=\sqrt{\frac{1}{2}\sum_{q=0,1}\sum_{m_k}\Pr[M_k=m_k\mid Q=q]\,\log_2\left[\frac{2\Pr[M_k=m_k\mid Q=q]}{\Pr[M_k=m_k\mid Q=0]+\Pr[M_k=m_k\mid Q=1]}\right]}$$

is the Jensen Shannon distance between the methylation probabilities $\Pr[M_k = m_k \mid Q = 1]$ and $\Pr[M_k = m_k \mid Q = 0]$ associated with the test and reference phenotypes, respectively.

2 Test statistic as distance metric

We would like the test statistic $T(q_1, q_2)$ we use for distinguishing between two phenotypes, q_1 and q_2, based on their methylation states within a genomic region of interest to satisfy the following properties: (i) $T(q_1, q_2) \geq 0$, for every q_1 and q_2 (non-negativity), (ii) $T(q_1, q_2) > 0$ if and only if $q_1 \neq q_2$ (positive definiteness), and (iii) $T(q_1, q_2) = T(q_2, q_1)$, for every q_1 and q_2 (symmetry). Non-negativity can always be satisfied by subtracting from a test statistic its minimum value. Positive definiteness is required to make sure that the test statistic takes its minimum value only when the two phenotypes are the same. Symmetry assures that the test statistic is the same irrespective of the order we use to compare two phenotypes.

In addition, we would like the test statistic to satisfy the following property (iv) $T(q_1, q_2) + T(q_1, q_3) \geq T(q_2, q_3)$, for every q_1, q_2, and q_3 (triangle inequality). To see why, let us assume that DNA methylation within a genomic region does not carry, on the average, sufficient information for distinguishing a phenotype q_1 from a phenotype q_2 as well as from a phenotype q_3. In this case, we also expect DNA methylation within the genomic region not to carry, on the average, sufficient information for distinguishing q_2 from q_3. Specifically, we expect that $T(q_1, q_2) \simeq 0$ and $T(q_1, q_3) \simeq 0$ implies $T(q_2, q_3) \simeq 0$, which is always true when T satisfies the triangle inequality.

Although the test statistic $1/K \sum_{k=1}^{K} [\text{JSD}(k)]^2$ we discussed in the Main paper satisfies properties $(i)-(iii)$, it does not satisfy property (iv). However, the test statistic given by Eq. (3) of the Main paper is a distance metric and, therefore, satisfies all four properties. This is a consequence of the fact that T is the Euclidean norm of the vector $(1/\sqrt{K})[\text{JSD}(1), \text{JSD}(2), \ldots, \text{JSD}(K)]$ of JSDs within GUs with data that overlap the genomic region, the fact that the JSD is a distance metric [2], and of the following lemma.

Lemma. Suppose that d_n, $n = 1, 2, \ldots, N$, are distance metrics and let

$$\boldsymbol{d}(\boldsymbol{x}, \boldsymbol{y}) := \begin{bmatrix} d_1(x_1, y_1) \\ d_2(x_2, y_2) \\ \vdots \\ d_N(x_N, y_N) \end{bmatrix},$$

where $\boldsymbol{x}$ and $\boldsymbol{y}$ are vectors with components x_n, y_n, $n = 1, 2, \ldots, N$. Moreover, let $||\cdot||$ be an absolute norm (i.e., a norm that is invariant to taking the moduli $|\cdot|$ of its components, which includes the Euclidean norm). Then $||\boldsymbol{d}(\boldsymbol{x}, \boldsymbol{y})||$ is a distance metric.

Proof. We must show that $||\boldsymbol{d}(\cdot, \cdot)||$ is non-negative, positive definite, symmetric, and satisfies the triangle inequality. Since a norm is always non-negative, $||\boldsymbol{d}(\boldsymbol{x}, \boldsymbol{y})|| \geq 0$, for every $\boldsymbol{x}, \boldsymbol{y}$, which proves non-negativity. Since a norm is also positive definite, we have that $||\boldsymbol{d}(\boldsymbol{x}, \boldsymbol{y})|| > 0$ if and only if $\boldsymbol{d}(\boldsymbol{x}, \boldsymbol{y}) \neq \boldsymbol{0}$, whereas, from the non-negativity of $\boldsymbol{d}(\boldsymbol{x}, \boldsymbol{y})$ and the positive definiteness of the d_n metrics, we have that $\boldsymbol{d}(\boldsymbol{x}, \boldsymbol{y}) \neq \boldsymbol{0}$ if and only if $x_n \neq y_n$, for $n = 1, 2, \ldots, N$, which proves positive definiteness. The symmetry property $||\boldsymbol{d}(\boldsymbol{x}, \boldsymbol{y})|| = ||\boldsymbol{d}(\boldsymbol{y}, \boldsymbol{x})||$ follows from the symmetry $d_n(x_n, y_n) = d_n(y_n, x_n)$, $n = 1, 2, \ldots, N$, of the d_n metrics. This leaves us to show the triangle inequality.

By using the fact that a norm satisfies the triangle inequality, we have that

$$\left|\left|\begin{bmatrix} d_1(x_1,y_1)+d_1(y_1,z_1) \\ d_2(x_2,y_2)+d_2(y_2,z_2) \\ \vdots \\ d_N(x_N,y_N)+d_N(y_N,z_N) \end{bmatrix}\right|\right| \leq \left|\left|\begin{bmatrix} d_1(x_1,y_1) \\ d_2(x_2,y_2) \\ \vdots \\ d_N(x_N,y_N) \end{bmatrix}\right|\right| + \left|\left|\begin{bmatrix} d_1(y_1,z_1) \\ d_2(y_2,z_2) \\ \vdots \\ d_N(y_N,z_N) \end{bmatrix}\right|\right|, \tag{S1}$$

for any $\boldsymbol{x}$, $\boldsymbol{y}$, $\boldsymbol{z}$. Moreover, any absolute norm is monotonic (see Theorem 2 in [1]). This means that $||\boldsymbol{a}|| \leq ||\boldsymbol{b}||$ for two vectors $\boldsymbol{a}$ and $\boldsymbol{b}$ such that $|a_n| \leq |b_n|$, for $n = 1, 2, \ldots, N$. Note now that the distance metrics d_n satisfy the triangle inequality, in which case, $|d_n(x_n, y_n) + d_n(y_n, z_n)| \geq |d_n(x_n, z_n)|$, for $n = 1, 2, \ldots, N$, which, together with the monotonicity property of $||\cdot||$, implies

$$\left|\left|\begin{bmatrix} d_1(x_1,y_1)+d_1(y_1,z_1) \\ d_2(x_2,y_2)+d_2(y_2,z_2) \\ \vdots \\ d_N(x_N,y_N)+d_N(y_N,z_N) \end{bmatrix}\right|\right| \geq \left|\left|\begin{bmatrix} d_1(x_1,z_1) \\ d_2(x_1,z_1) \\ \vdots \\ d_N(x_N,z_N) \end{bmatrix}\right|\right|.$$

This result, together with Eq. (S1), shows that $||\boldsymbol{d}(\cdot,\cdot)||$ satisfies the triangle inequality. ♠

Note finally that $0 \leq T \leq 1$, since the JSD is always a number between 0 and 1 [2].

References

1. F. L. Bauer, J. Stoer, and C. Witzgall. Absolute and monotonic norms. *Numer Math*, 3: 257–264, 1961.

2. D. M. Endres and J. E. Schindelin. A new metric for probability distributions. *IEEE Trans Inform Theory*, 49:1858–1860, 2003.

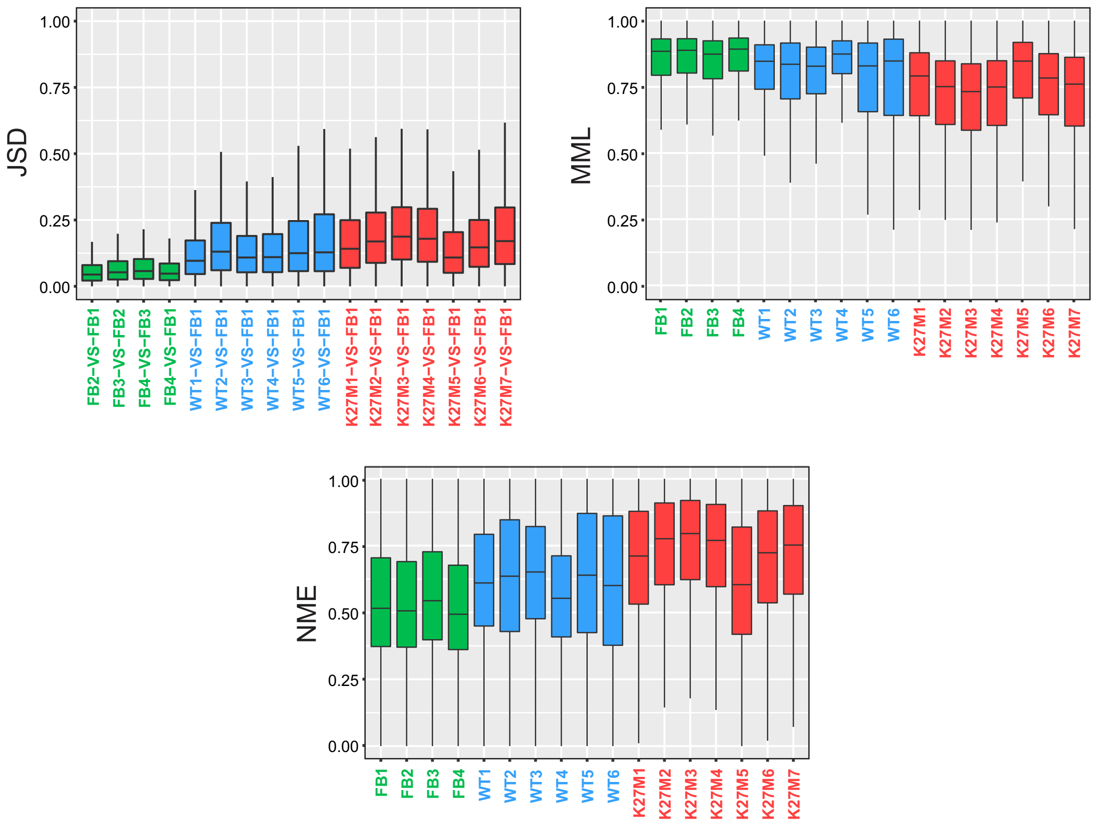

Figure S1. Boxplots of genome-wide distributions of JSD, MML, and NME values in the normal fetal brain, H3.3-WT, and K27M mutant samples considered in this paper. The JSD values show small methylation discordances associated with the fetal brain samples (which are due to biological, statistical, and technical variability), thus confirming their appropriateness as normal controls. Moreover, the JSD demonstrates a global increase in methylation discordance within the tumor samples, accompanied by global hypomethylation (MML) and gain in methylation entropy (NME) in most samples. Center lines: median; boxes: interquartile range (IQR); whiskers: $1.5 \times$ IQR.

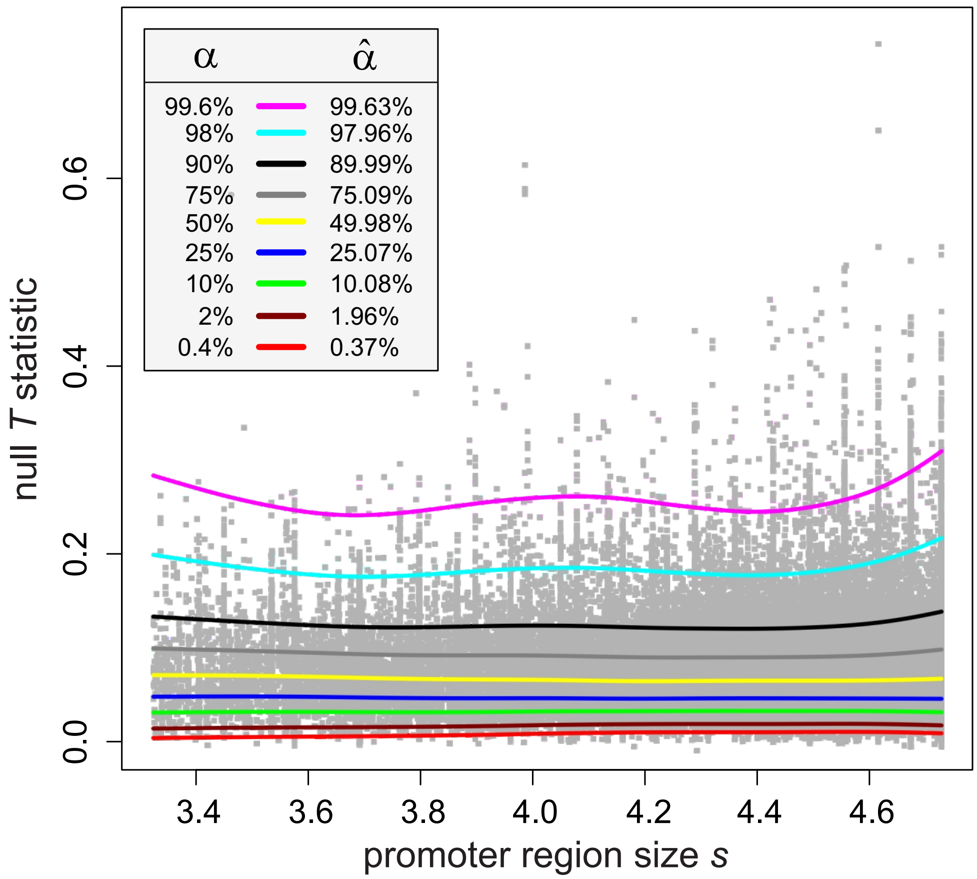

Figure S2. α-centile curves, calculated for different values of α from the estimated logitSST-based null PDF $\widehat{f_0}(t;s)$ within promoter regions, drawn over a scatter plot of 104,694 observed pairs (t_k, s_k) of null T statistic values t_k and promoter region sizes s_k. The percentage $\widehat{\alpha}$ of empirically observed data points that fall below a centile curve agrees well with the corresponding α value, indicating that $\widehat{f_0}(t;s)$ is consistent with the data.

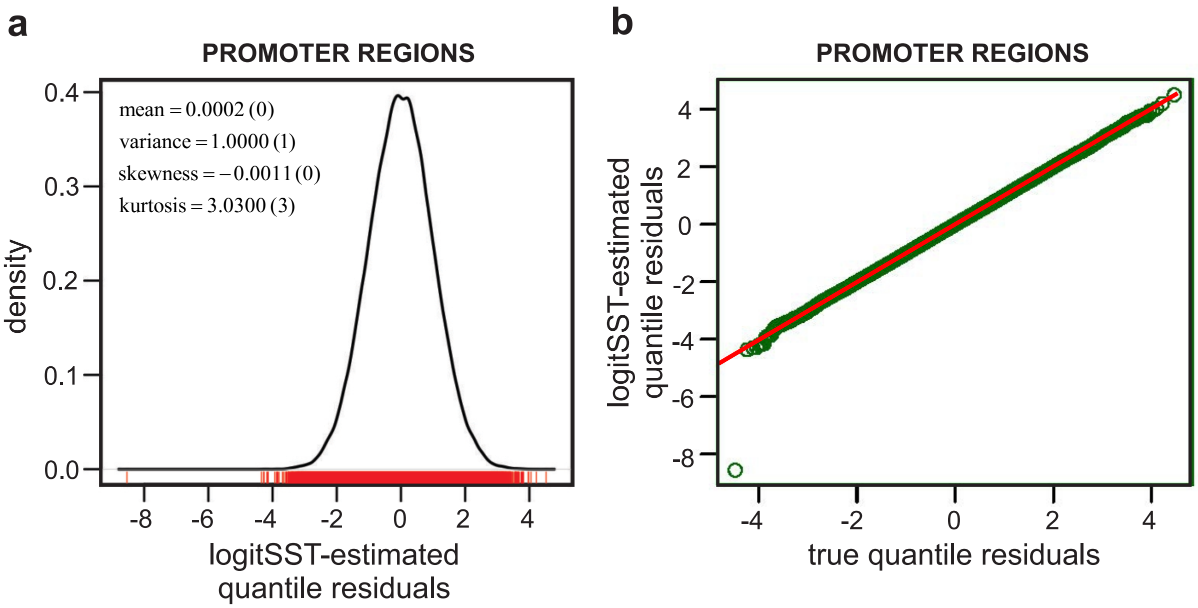

Figure S3. Quantile residual analysis of the logitSST-estimated null PDF of the T statistic in the case of promoter regions. (a) The kernel density approximation of the distribution of the logitSST-estimated quantile residuals (bottom red marks) demonstrates close agreement with standard normality. (b) The Q-Q plot (green marks) of the logitSST-estimated quantile residuals against the corresponding true quantile residuals is very close to the diagonal (red) line, suggesting a close agreement of the logitSST-estimated null PDF of the T statistic to its true distribution.

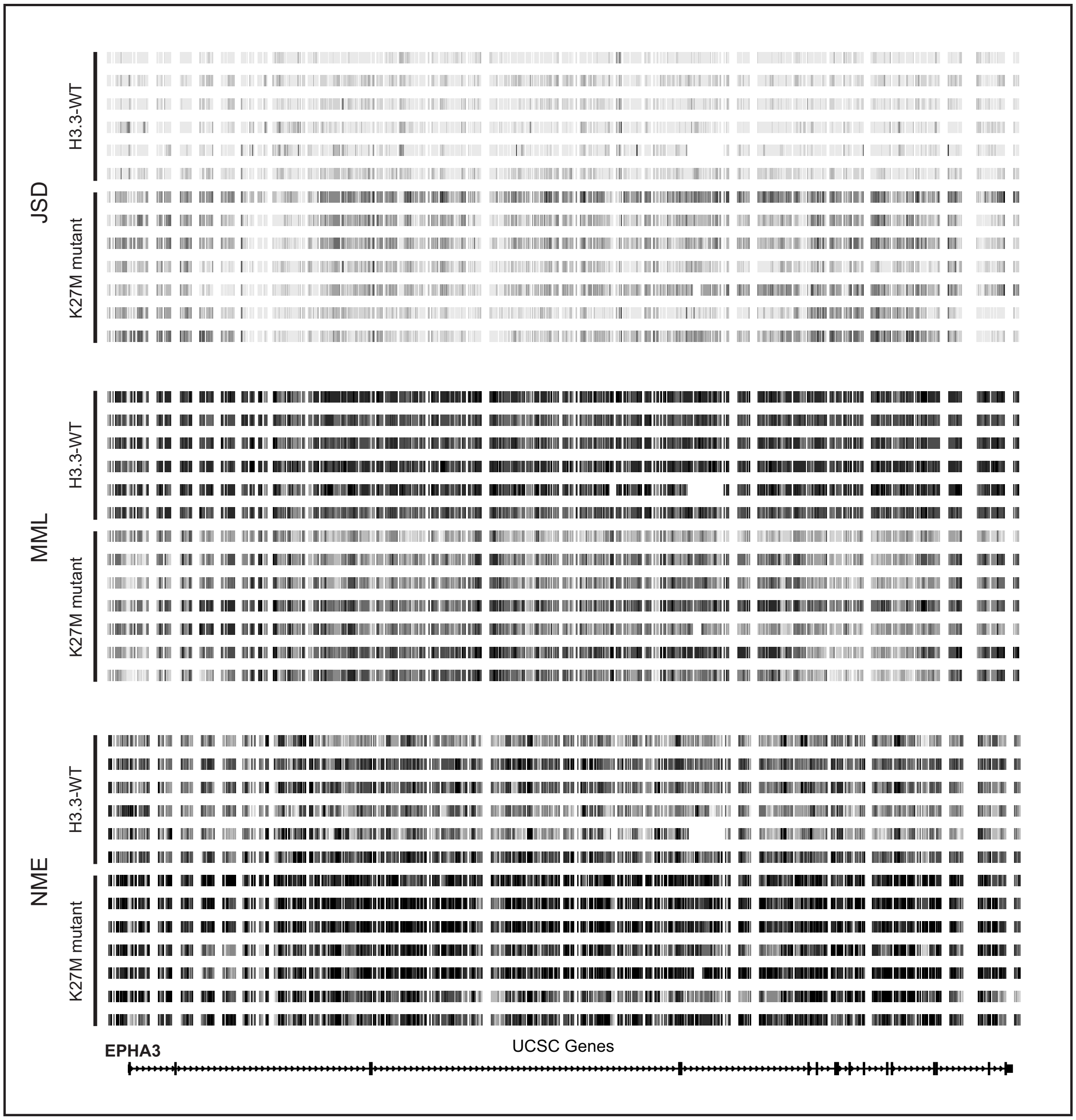

Figure S4. UCSC genome browser images of JSD, MML, and NME tracks within the genomic region [chr3: 89,145,180–89,536,200] that contains *EPHA3*, obtained by informME in the six H3.3-WT vs. FB1 and the seven K27M mutant vs. FB1 comparisons. Light colors indicate small values, whereas dark colors indicate large values. The JSD tracks demonstrate increased methylation discordance in the K27M mutant samples, which is associated with widespread hypomethylation (MML tracks) and a gain in methylation entropy (NME tracks) close to its maximum value.

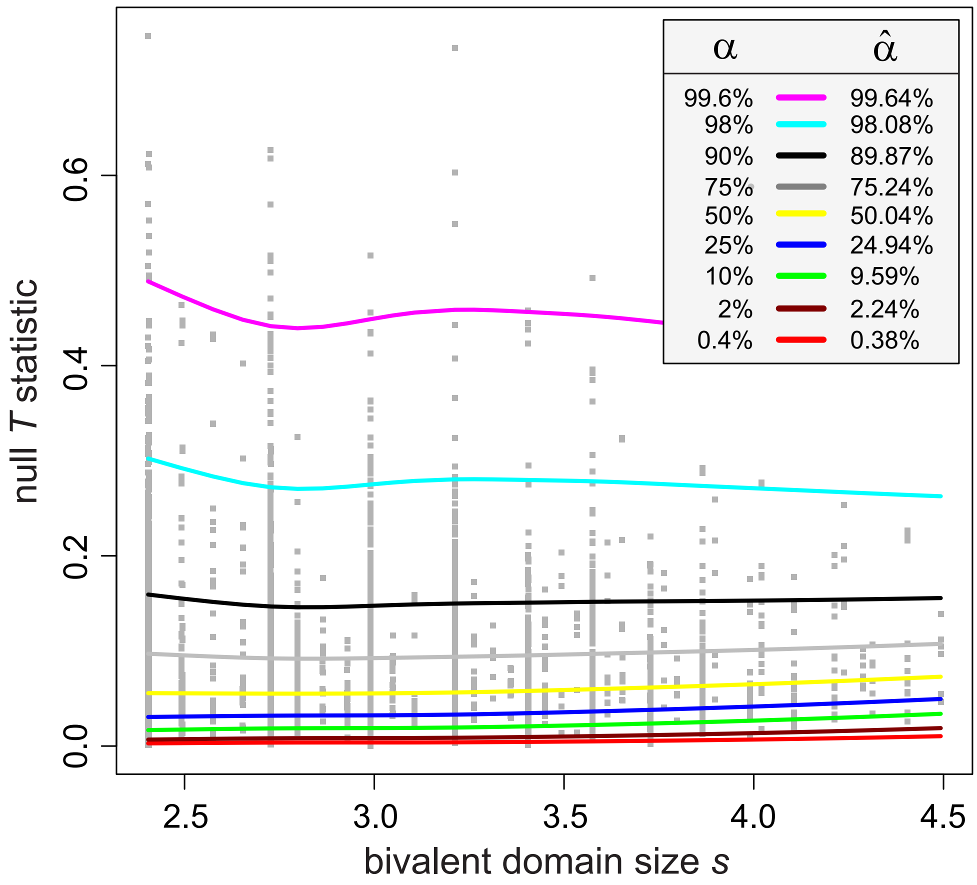

Figure S5. α-centile curves, calculated for different values of α from the estimated logitSST-based null PDF $\widehat{f_0}(t;s)$ within bivalent domains, drawn over a scatter plot of 7,446 observed pairs (t_k, s_k) of null T statistic values t_k and bivalent domain sizes s_k. The percentage $\widehat{\alpha}$ of empirically observed data points that fall below a centile curve agrees well with the corresponding α value, indicating that $\widehat{f_0}(t;s)$ is consistent with the data.

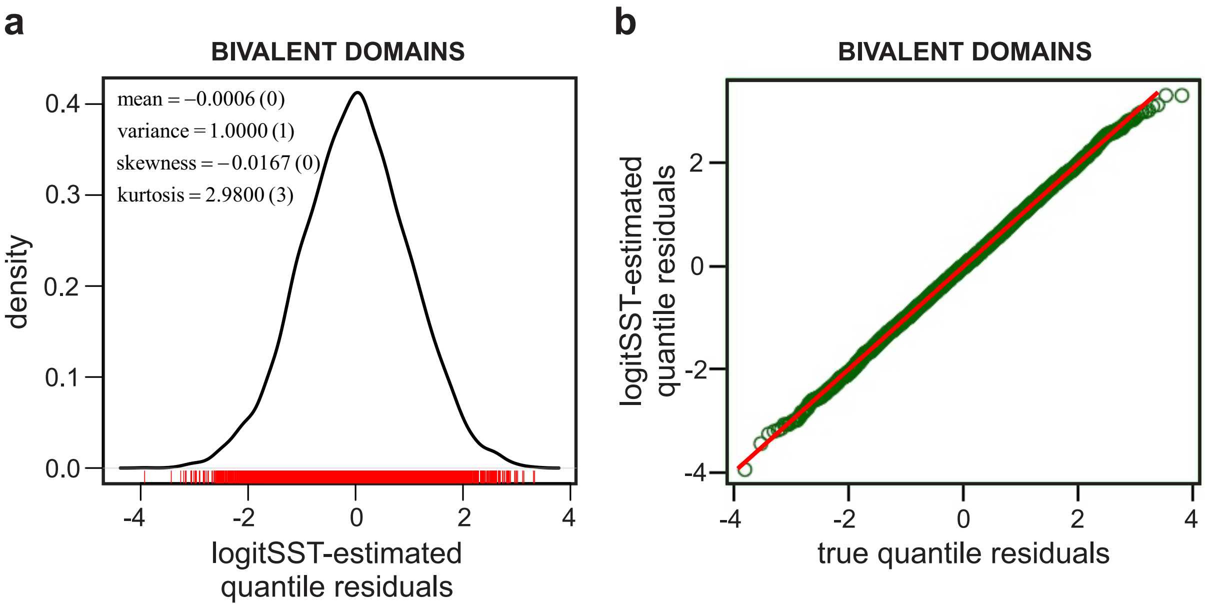

Figure S6. Quantile residual analysis of the logitSST-estimated null PDF of the T statistic in the case of bivalent domains. (a) The kernel density approximation of the distribution of the logitSST-estimated quantile residuals (bottom red marks) demonstrates close agreement with standard normality. (b) The Q-Q plot (green marks) of the logitSST-estimated quantile residuals against the corresponding true quantile residuals is very close to the diagonal (red) line, suggesting a close agreement of the logitSST-estimated null PDF of the T statistic to its true distribution.
